# Immune Responses Elicited by Outer Membrane Vesicles of Gram-Negative Bacteria: Important Players in Vaccine Development

**DOI:** 10.3390/life14121584

**Published:** 2024-12-02

**Authors:** Branko Velimirov, Branko Alexander Velimirov

**Affiliations:** Division of Microbiology and Molecular Biology, Medical Faculty, Private Sigmund Freud University, Freudplatz 3, 1020 Wien, Austria; branko.a.velimirov@med.sfu.ac.at

**Keywords:** bacterial antigens, outer membranes, immune response, Toll-like receptors, antimicrobial peptides, vaccine platform

## Abstract

The attractiveness of OMVs derived from Gram-negative bacteria lies in the fact that they have two biomembranes sandwiching a peptidoglycan layer. It is well known that the envelope of OMVs consists of the outer bacterial membrane [OM] and not of the inner one [IM] of the source bacterium. This implies that all outer membranous molecules found in the OM act as antigens. However, under specific conditions, some of the inner membrane proteins can be exported into the outer membrane layer and perform as antigens. A key information was that the used purification procedures for OMVs, the induction methods to increase the production of OMVs as well as the specific mutant strains obtained via genetic engineering affect the composition of potential antigens on the surface and in the lumen of the OMVs. The available literature allowed us to list the major antigens that could be defined on OMVs. The functions of the antigens within the source bacterium are discussed for a better understanding of the various available hypotheses on the biogenesis of vesicle formation. Also, the impacts of OMV antigens on the immune system using animal models are assessed. Furthermore, information on the pathways of OMVs entering the host cell is presented. An example of a bacterial infection that causes epidemic diseases, namely via *Neisseria meningitidis*, is used to demonstrate that OMVs derived from this pathogen elicit protective immune responses when administered as a vaccine. Furthermore, information on OMV vaccines under development is presented. The assembled knowledge allowed us to formulate a number of reasons why OMVs are attractive as vaccine platforms, as their undesirable side effects remain small, and to provide an outlook on the potential use of OMVs as a vaccine platform.

## 1. Introduction

Bacterial outer membrane vesicles belong to the category of extracellular vesicles (Evs). They transport signal molecules over long distances [1,2]. These spherical structures [20 to 250 nm in diameter], often containing a high amount of membrane proteins, enable and facilitate intercellular interactions [3,4,5,6] and have been observed in Gram-negative and Gram-positive bacteria [7,8] and archaea [9]. OMVs, which derive from the outer membrane of Gram-negative bacteria, are generally composed of those bacterial proteins, lipids and glycans that are found in the outer membrane of the parental cells. Furthermore, they may contain periplasmic molecules such as nucleic acids [10,11,12,13,14,15,16] and specific enzymes or peptidoglycan molecules in their lumen. Therefore, the idea to use OMVs from bacterial pathogenic strains as vaccines against their parental strains was a logical consequence [17]. In the same line of consideration, the possibility to create drug delivery systems by loading OMVs with specific cargo emerged [17,18]. Within the last 25 years, OMVs were tested by various research groups as cheap alternatives for vaccines, and promising results were obtained in animal test models [19,20]. Since OMVs are non-replicative as compared to their pathogenic bacterial parent strains, it is expected that their antigen load elicits an adequate immune response of the host as long as the applied OMV concentration is compatible with the host’s physiological status.

In the following treatise, the major aim is to present the state of the art with respect to the use of OMVs in complementing or replacing classical vaccines. This implies that (1) it will be necessary to attempt an overview of the potential antigens presented by OMVs. To this end, information on the mechanisms leading to OMV formation is needed whereby both advantages and disadvantages of the OMV composition of the various vesicles to be used as vaccine platforms shall be discussed; (2) the effects of these antigens on the immune system will be mentioned; and (3) the vaccine potential of OMVs based on the assembled knowledge from literature will be discussed.

## 2. Biogenesis of OMVs

### 2.1. Membrane Structure

**Figure 1:**
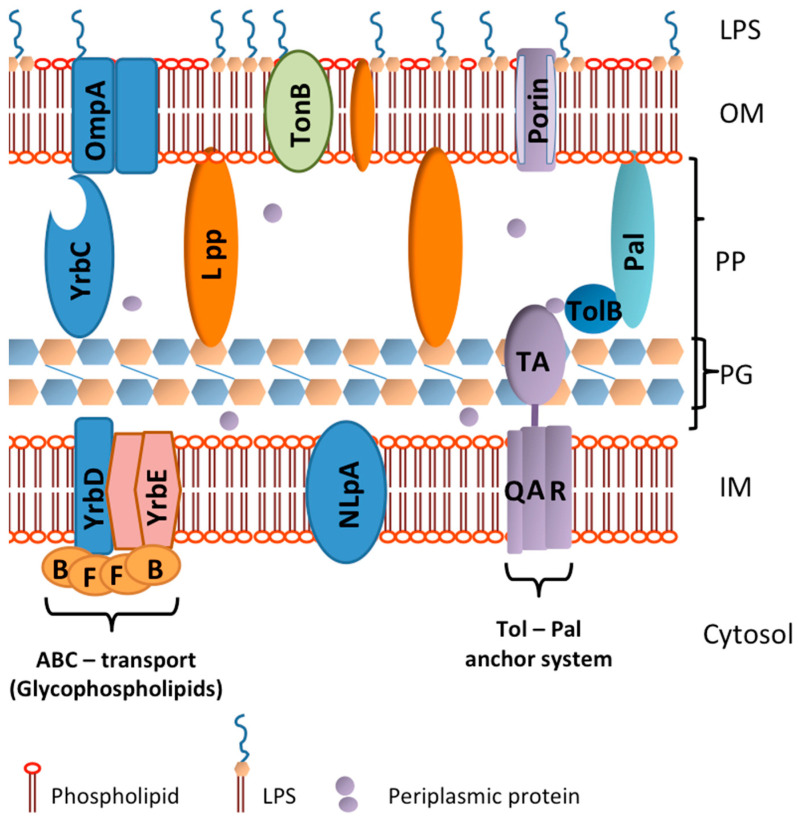
Cell envelope of a hypothetical Gram-negative bacterium: The bacterial outer membrane (OM) is composed by an outer leaflet with an asymmetric distribution of lipid components due to the presence of lipopolysaccharides (LPS); the inner leaflet is composed of glycerophospholipids. Both leaflets of the inner membrane (IM) are symmetrical with respect to the phospholipids and contain protein complexes such as the Tol-Pal anchor system, NlpA or the ABC-transport system YrbDE. The OM contains a number of proteins (Porin and TonB), lipoproteins (Lpps) and the outer membrane protein OmpA that connects the ABC transport system. The space between the IM and OM is the periplasmatic space (PP) and contains the peptidoglycan layer (PG) as well as members of the YrbDE protein complex, namely YrbC. The white open pouch like the YrbC part indicates that it is a region where molecules can bind for transport. The molecules YrbB and YrbF edge the inner leaflet of the IM. The PP is dominated by both PG and the connecting Lpps and the TolB and Pal molecules with the YrbC and periplasmic proteins.

The unique membrane structure of Gram-negative bacteria consists of an outer and an inner membrane, a peptidoglycan layer and a periplasm, whereby the outer leaflet of the outer membrane contains both membrane proteins and lipopolysaccharides [LPS]. The inner membrane displays mainly phospholipids. The peptidoglycan layer in the periplasm connects to the outer and inner membrane by means of outer membrane proteins like OmpA or Lpps (Figure 1). There are several steps in the formation of OMVs. To start the process, the protein linkers have to detach, which implies that the binding between peptidoglycan and outer membrane is lost.

This disconnection permits the liberation of the outer membrane and the formation of a vesicle. During this process, the outer membrane proteins, such as OmpA, are integrated in the OMVs. However, the formation of OMVs cannot be explained by this short description. When screening the literature for more detailed information on the formation process of OMVs, it becomes obvious that (1) there is a variety of hypotheses explaining OMV formation [21,22,23,24,25,26], but it seems that (2) a number of offered hypotheses are species specific and it cannot be excluded that many of the described mechanisms work together for the formation of OMVs and that (3) there are different induction methods developed to increase OMV production that shed new light on both the formation mechanism of OMVs and their use as antigen delivery for vaccine purposes.

### 2.2. Hypotheses on the Mechanisms of OMV Formation

A number of discoveries allow speculation of the elaborate mechanisms driving the biogenesis of OMVs [1,27]. A simple imbalance between bacterial growth and OM biosynthesis could induce a release of excess membrane material which would re-establish the material balance within the bacterium and result in OMV formation.

Zhou et al. [28] showed that in *Porphyromonas gingivalis*, OMV production was connected to cell wall turnover. The turgor pressure on the OM during cell wall synthesis exerted by muramic acid and peptidoglycan induces the induction of blebs. The release of pinched-off blebs as OMVs relieves the cell from the excess pressure.

Environmental stress exerted on a Gram-negative bacterium is first felt on the OM of the cell and frequently results in OMV production. Such situations were obtained via toxic concentrations of long-chain alcohols, EDTA, osmotic pressure and heat shock on *Pseudomonas putida* DOT-T1E [29] and *E. coli* [30]. It was forwarded that temperature stress leads to the accumulation of misfolded proteins which are then removed from the cell via OMVs. Increased OMV production was also obtained when administering antibiotics to bacterial cell cultures. Antibiotic stress on *Pseudomonas aeruginosa* [31] induced by ciproflaxin leads to an SOS response as soon as DNA replication is inhibited. This SOS response was assumed to contribute to increasing OMV production. An additional and important aspect of OMV formation in *P. aeruginosa* deals with the role of PQS [Pseudomonas quinolone signal] in OMV formation [32,33,34]. This quorum-sensing signal stimulates not only virulence genes but has been described as a multifunctional molecule altering transcriptional profiles of genes, mediating iron acquisition, cytotoxicity, host immune responses and OMV biogenesis. PQS integrates into the outer membrane and induces membrane curvature [32,33,35], thus promoting OMV formation.

An association between the appearance of OMVs and the flagellated source bacteria (versus nonflagellated cells) was suggested from several authors [36,37,38]. The flagellin protein (FliC), detected in OMVs from *E. coli* via proteomic analysis [37], led to the assumption that flagellae may influence vesiculation. Follow-up experiments with hyperflagellated swarmer strains from *V. fischeri* and nonflagellated *flrA* mutants indicated high versus low vesiculation rates, respectively [36]. Within this same study, which included vesiculation comparisons with *E. coli* and *V. parahaemolyticus*, the authors concluded that species expressing flagellae released more vesicles than nonflagellated species. Flagellae and flagellar movement may perturb the outer membrane, thereby promoting OMV production. The question remains on whether flagellar movement induces OMV release from the near surrounding of the flagellum site or whether the whole bacterial membrane is affected [39]. Moreover, this could imply that fast-moving bacteria, which have more rigid and complex flagellae (Manson et al., 1998 [40]; Lengler, Drews and Schlegel, 1999 [41]) moving along a concentration gradient to reach an attractant, have a higher potential to produce OMVs as compared to resting strains. It is remarkable that some Vibrio, Serratia and Proteus species switch their morphology periodically from short and sparsely flagellated cells to elongated and highly flagellated cells. To which extent this phenomenon impacts OMV production needs to be investigated.

An important contribution in understanding OMV formation was provided by Kitigawa et al. [42], in emphasizing the importance of proteins in vesiculation. It was demonstrated that proteins play an essential role in OMV biogenesis of *Salmonella enterica*. Biogenesis was provoked by inducing the *Salmonella*-specific protein PagC, whose expression is activated by conditions mimicking acidified macrophage phagosomes. An increased expression of this protein also increases vesiculation, and PagC was found to be an important constituent in *Salmonella*-derived OMVs. Similarly, the protein NlpA, which is a protein of the inner membrane of *E. coli*, also contributes to regulating OMV biogenesis [43]. The majority of molecules with tethering functions in stabilizing the IM–OM relation via the intermediate of the PG are also involved in triggering OMV formation, which are either protein complexes or lipoproteins. Braun’s lipoprotein (BLpp) in *E. coli* is the only covalent tether between the OM and PG [44]. However, most Gram-negative bacteria lack BLpp and display different Lpps. This led to the assumption that alternative mechanisms of OM stabilization exist [45,46] and that disruption of tethering molecule complexes induces vesicle formation. Examples include proteins of the Tol-Pal complex, the YrbC, YrbF and YrbB proteins as well as associated VacJ and OmpA proteins. Sandoz et al. [46] showed that ß-barrel outer membrane proteins such as BbpA are covalently attached to PG in Gram-negative bacteria like *Coxiella burnetii*, *Agrobacterium tumefaciens* and *Legionella pneumophila*, in which disruption would remove cell envelope stabilization and provoke OMV formation. For *P. aeruginosa*, a model for vesicle formation was proposed by Kadurugamuwa and Beveridge [23] and subsequently confirmed by observations from Sabra et al. [22]. This bacterial species is composed of two chemically and antigenically clear, distinct forms of LPS. It can produce LPS with either A-band O-specific polysaccharides, which are electroneutral, or else B-band O-specific polysaccharides, which are negatively charged [47]. Those cells with electronegative charges of the B-band LPS tend to cause charge-to-charge repulsion [48,49] and instability of the OM. This will result in membrane blebbing and OMV formation as well as trapping of neighboring periplasmic components within the OMVs. Unfortunately, the authors [48] do not discuss how OMV formation is to be understood in *P. aeruginosa* for LPS with A-band O-specific polysaccharides, which is the more conserved form in most *Pseudomonas* species.

The above presented information reinforces the fact that there is presumably not a single formation mechanism inducing OMV formation and that we have to expect that several formation processes do interact. A surprising observation that merits mentioning is that for certain bacterial species like *Delftia* sp. Cs1-4, a soil bacterium [50], and *Francisella novicida*, considered a rare pathogen [51], the appearance of OMVs was recorded without forwarding a formation hypothesis. Instead, the authors concentrated on the mode of OMV delivery. The appearance of tube-like structures was observed projecting from the bacterial cell surface and releasing OMVs into the environment. So far, we have no indications that similar observations were made for pathogenic Gram-negative bacteria, and it seems that this phenomenon occurs in cells living in aquatic or soil environments.

### 2.3. Toxins and Antigens on OMVs

Molecules that are found on the membranes of OMVs may be both useful as antigens or else harmful, as they have toxic effects on the host. It is known that OMVs act as delivery vesicles for bacterial toxins into host cells [20,52,53,54,55]. Among these molecules, we group [a] bacterial virulence factors, such as endotoxins and exotoxins, whereby there are numbers of toxin subclasses according to the their targets in the host; genotoxins such as the cytolethal distending toxin (CDT), expressed by a number of Gram-negative bacteria such as *Aggregibacter actinomycetemcomitans,* which is a holotoxin, since it is assembled of subunits [55], and an exotoxin that is able to inhibit T and B cells. Furthermore, virulence factors, such as enterotoxins, as well as neurotoxins, leukocidins and hemolysins are well investigated [56]. OMVs from enterotoxigenic *E. coli* [ETEC] and enterohemorrhagic *E. coli* [EHEC] are known to transport the enterotoxin LT [52] and the Shiga toxin [57] characteristic for *Shigella dysenteriae* serotype 1, respectively. The toxin of *V.cholerae* is a strong enterotoxin [an A/B hollotoxin] causing severe gastrointestinal symptoms. The pertussis toxin, which is an ADP-ribosyltransferase-targeting G-protein [58] and Listeriolysin O toxin, a pore-forming toxin, need to be mentioned [58,59]. Additionally, OMVs display adhesion factors, invasion factors and antiphagocytic factors.

The LPS endotoxin is considered as the most potent factor for immune stimulation delivered by OMVs [60,61], which led to two different concepts of thought. On the one hand, the observed density of LPS on OMVs was regarded as too dangerous when used as vaccines, as a systemic reactogenicity of the lipid A on humans may be harmful. On the other hand, the presence of LPS on OMVs provides an essential support to elicit optimal immune stimulation. Therefore, a key factor for the design of OMV-based vaccines is to achieve a balance between the risk of triggering reactogenicity and efficient stimulation of an adequate immune response [62]. For this purpose, the necessity to manipulate the structure of lipid A becomes obvious. The lipid A part of LPS, consisting of the ß-1,6-linked disaccharide glucosamine that is phosphorylated at the 1 and 4’ positions and acylated with six chains of fatty acids (differing in length and saturation level), is the most reactogenic scaffold form. Its conserved molecular pattern is the major inducer of immunological response to LPS. The recognition of LPS is due to the TLR 4–MD-2 heterodimer. Two accessory proteins, namely the LPS-binding protein and CD14, enable the extraction of LPS from the bacterial membrane and the transfer to the TLR 4–MD-2 complex. Extensive studies on the structure–activity relationship of lipid A of LPS [63] have shown that the most important factor in governing immunological activity of LPS is the number of lipid chains of lipid A. Optimal inflammatory activity is obtained with six chains, while five chains in lipid A are a hundredfold less active [63]. An effective approach to modify the status of the acyl chains functions is via the deletion of genes encoding for HtrB, MsbB and MsbP PagP in *Shigella* [62,64], *Vibrio* (Leitner et al. 2013 [65]) and *Salmonella* [66], respectively. This results in OMVs with a dominance of penta-acylated lipid A.

Another way to reduce endotoxic activity of LPS is the deletion of one or both phosphate groups of lipid A (Ref), which could be accomplished by knocking in the lpxE gene encoding for the dephosphorylase in *Francisella tularensis* [67].

As for [b] proteins with antigen functions, we distinguish between those derived from the outer membrane and the inner membrane layers. Most of the proteins (irrespective whether they are linked to glycans or lipids) stem from the outer bacterial membrane (Figure 1), but proteins from the inner layer may also be found on OMVs, depending on the experienced stress situation to which the strains are exposed and the applied induction method to increase vesiculation (see below). Among the outer membrane proteins, like porines, both Omp F and the Por A protein are very immunogenic as well as OmpA in *E. coli*, Omp 22, 25 and 31 found in *Brucella abortus*, *Brucella* sp. and *Acinetobacter baumannii* [68,69,70]. An example for immunogenic membrane receptors is Ton B, which serves as an iron transporter by means of siderophores. A well-investigated enzyme that elicits humoral and cellular immune responses is Htra [rHtra]. The function of this high-temperature requiring serine protease was recently documented in *Campylibacter jejeuni* [71], in pathogenic *E. coli*, *Helicobacter pylori* and *Shigella flexneri* [72,73]. Potential antigens are protein complexes such as the YrbFEDB or proteins of the Tol-Pal system. The central part of the YrbFEDB protein complex, which is an ATP-binding cassette [ABC] transporter, namely YrbED, is situated in the inner membrane of Gram-negative bacteria and can contact with OmpA via YrbC and VacJ (a lipoprotein), while YrbB and YrbF are situated on the outer side of the inner membrane [74] (Figure 1). YrbE is a transporter for phospholipids [75,76]. As these protein complexes are anchored in the inner membrane, they will not appear on spontaneously formed OMVs, but specific vesicle induction methods may transport some of these inner membrane proteins, including NlpA, to the outer membrane, and hence on the OMVs, where they display antigen properties.

The Tol-Pal protein system, being essential for enzymes to cleave the PG in order to complete division of bacterial cells [77], is also situated in the inner membrane and will appear in the OM of the vesicles in similar situations as for the abovementioned YrbE phospholipid transporter proteins.

The factor H-binding protein [FHbp], being important for the survival of *Neisseria* species in host blood, is also known to be involved in response to stress to avoid accumulation of misfolded proteins [78]. It is highly immunogenic and is a major target for antibodies, as it is surface expressed by nearly all *Neisseria* strains causing diseases [79]. Additionally, lipidated FHbp were shown to induce superior immune responses compared to the non-lipidated form.

The immunogenic effect of lipoproteins [Lpps], such as Brauns Lpp from *E. coli* or any tethering lipoprotein between the inner leaflet of the OM and the peptidoglycan layer [such as Nlp1], was repeatedly observed [80,81]. The recognition of bacterial peptidoglycans by the innate immune system is not always evident, as the molecular diversity within peptidoglycans may vary between species [82]. Thus far, it has been shown that the presence of peptidoglycans is recognized by receptors on cells of the innate immune system, inducing inflammatory responses [83].

## 3. Toll-Like Receptors [TLR] and Pathogen-Associated Molecular Patterns [PAMPs]

Many of the molecules that act as antigens and decorate OMVs act as activators of Toll-like receptors [TLRs], thus eliciting often powerful immune responses. These TLRs, which belong to the pattern recognition receptors [PRRs], are characteristic for macrophages, neutrophils, B-cells and dendritic cells, but are also found on epithelial cells and fibroblasts. These receptors recognize pathogen-associated molecular patterns [PAMPs] as well as damage-associated molecular patterns [DAMPs], which are derived from damaged host cells or from the residues of dead cells [84,85,86,87,88]. In addition to the well-investigated TLRs, other receptor classes are known, namely NOD-like receptors [NLRs], which are intracellular molecules, while TLRs are transmembrane receptors [82]. RIG-I-like receptors [RLRs] are also transmembrane receptors and cytosolic sensors for DNA, being able to react with different classes of PAMPs [89,90].

TLRs are membrane glycoproteins and consist of two domains, an extracellular domain with leucine rich repeats and a cytoplasmic Toll/interleukin-1 receptor [TIR]. The extracellular domain serves for PAMP recognition, and the TIR domain is needed for downstream signaling [91]. Adaptor molecules [MyD88, TRIF, TIRAP, TRAM], recruited to the TIR domain, activate transcription factors like MAP kinases, IRF 3/7 and NF-kappaB, resulting in the production of pro-inflammatory cytokines and type I interferons; this initiates and shapes the adaptive immune response. Note that TLR 1–TLR 10 have been identified in humans, while TLR 1–TLR 9 and TLR 11–TLR 13 have been identified in mice. Among the TLRs, we distinguish surface receptors and those that are expressed intracellularly. Surface receptors respond to bacterial PAMPs [i.e., components of the microbial membrane] and PAMPs on fungi and protozoa. Bacterial and viral nucleic acids are recognized by intracellular TLRs [84,92].

As OMVs represent with minor modifications the bacterial outer membrane, they are composed of a large array of bacterial antigens, including the molecules encapsulated in their lumen; these are mostly periplasmic proteins, occasionally flagellin [87,88,89] as well as ribosomal RNA and ss or dsDNA fractions [10,11,13,14]. The PAMPs of the majority of these antigens bind on seven receptors, of which two are endocytic [TLR 9 and TLR 13] and five are surface receptors [TLR 1, TLR 2, TLR 4, TLR 5 and TLR 6]. TLR 1 can dimerize with TLR 2 or TLR 6 and recognizes bacterial lipoproteins; TLR 4 in combination with myeloid differentiation 2 [MD-2] form a receptor complex, recognizing the lipid A domain of LPS where LPS-binding protein [LBP] is involved [63,93]. TLR 5 reacts with flagellin, and TLR 9 and TLR 13 recognize unmethylated CpG and ribosomal RNA, respectively [94,95]. OMVs with dsRNA load were recognized from TLR 3; in the case of ssRNA, cargo recognition by TLR 7 was recorded [96].

### PAMPs-Induced Immune Responses

The activation of the immune system by TLRs due to the presence of PAMPs on OMVs is closely related to a key process [97,98,99], namely the maturation of dendritic cells [DCs] of the innate immune system. This process is marked by an increase and accelerated distribution of surface MHC class II and co-stimulatory molecules [like CD80, CD86]. The maturation of DCs, in turn, initiates antigen-specific immune responses and enables the priming of T cells, leading to both activation and survival of T cells. This process is also driven by the fact that the co-stimulatory molecules function in tandem as ligands for CD28 and CTLA-4 [cytotoxic T-lymphocyte-associated Protein 4], which act as downregulators of the immune response. MHC class I proteins, which are expressed on the surface of all cells with nuclei and are also synthesized in the endoplasmic reticulum, present foreign particles to zytotoxic T cells, provoking an adaptive immune response.

Different effector responses will occur when DCs recognize a PAMP. These responses will depend on the stimulated receptors as well as on the released cytokines. Thus, an unprimed T cell may transform into a Th1, Th2 or Th17 cell, depending on which TLR is presenting the antigen and on cytokine release from the antigen-presenting cells. Examples for various effector responses are *H. pylori*-derived OMVs [100], which induce the release of IL-8 in epithelial cells. This was also observed for *Pseudomonas aeruginosa* [101], and it is worth mentioning that the IL-8 release triggers recruitment of monozytes and neutrophiles.

For *S. typhyrimurium*-derived vesicles, a significant increase in MHC II and CD86 on macrophages and DCs was recorded [67] as well as an increase in IL-12 and TNF-alpha. As DCs are heterogeneous with respect to their function, a classification was proposed according to the expression of class-specific PRRs [102,103]. Therefore, TLR activation by exogenous stimuli is also dependent on the DC class that is activated.

A special class of DCs are plasmacytoid dendritic cells [pDCs], which secrete high levels of type I interferons [IFN alpha and beta]. They are found in various peripheral organs and derived from pre-DC populations and sensors for viral infections via TLR 7 and TLR 9. In the following, specific signaling pathways are induced, leading to the genesis of specific cytokines that determine which Th-cell-subset will be produced [103]. A number of studies on OMVs from *V. cholerae*, *S. thyphimurium*, *B. burgendorfi* and *Flavobacterium* sp. [19,104,105,106] indicated that a combination of LPS with the bacterial OM generated effective immune responses. The importance of TLR activation on the maturation of phagosomes and DCs was demonstrated, and antigen presentation on MHC I and MHC II was noted as well as B cell responses. A different subset of DC cells, the CD8a+ DCs, was recognized to be relevant for the activation of CD8+ T cells [67]. These DCs present both Rig-like receptors (RLRs) and TLR 3; they produce IL -12 and type I IFNs [Schulz et al. 2005 [107]. In this context, it is worth mentioning that for neoantigens (peptide antigens encoded by tumor-specific mutated genes) expressing tumor cells, it could be shown that treatment with OMVs from *E. coli* (Won et al. 2023 [108]) increased activation and infiltration of CD8+ T cells, especially of those with high expressions of TCF-1 and PD-1.

These observations are additional indications that OMVs are able to elicit effective antigen responses due to antigen delivery. Despite the potential therapeutic applications, the low yield of normal or spontaneous OMV formation retards experimentation, and large-scale tests for the development of OMVs as vaccines or vaccine complements are needed. Therefore, many efforts have been made in the last decades to increase vesiculation processes in bacterial cultures.

## 4. Production of OMVs

### 4.1. Induction and Yield Increase in OMVs for Antigen Presentation

Irrespective of the fact that there is no single OMV formation hypothesis, which is generally accepted, there is a need to produce large quantities of OMVs within a limited time in order to enable experimental tests with respect to specific antigen presentation and vaccine development. The spontaneously formed OMVs from all Gram-negative bacteria [sOMVs] display therapeutic effects [109,110,111], but even in the logarithmic phase the harvestable quantity of sOMVs is by far too low. Therefore, it was decided to use sOMVs as standard vesicles, which could be used to compare OMVs obtained by various strategies to increase OMV production in order to describe qualitative differences between OMVs.

### 4.2. Mechanically Disrupted Bacteria [lOMVs]

A number of methods have been developed to obtain hypervesiculation of OMVs such as mechanical disruption of the bacterial cells [112] via sonication or vortexing [lOMVs]. Sonication or vortexing of the bacterial pellets leads to the fragmentation of the cells and the subsequent fusion of the fragments, which forms harvestable OMVs. The disadvantage of this approach is the inclusion of undesirable non-membrane components into the OMVs [113], which increase antigenicity but decrease safety and is unsuitable for vaccine development.

### 4.3. OMVs Induced by Extraction Molecules [nOMVs]

Destabilization of bacterial membranes via EDTA has frequently been used to increase the OMV harvest [114]. This chelating agent is known to remove calcium ions from the surrounding environment and thereby affects bacterial membranes. As calcium ions neutralize the repelling negative charges of LPS [115] as well as other anionic lipids, a repelling effect between LPS molecules results in destabilized membranes. Consequently, a yield increase in OMVs is obtained [48]. As the method is less aggressive than mechanical disruption, the OMVs retain the LPS and the molecular cargo of the OM and the lumen. Even though they are better suited for vaccine development than lOMVs and dOMVs, they have the disadvantage that the removal of the calcium ions is expected to induce the formation of unstable OMVs.

### 4.4. Detergent-Induced OMVs [dOMVs]

A frequently used method to increase the OMV harvest is the application of detergent extraction. Bacteria were exposed to detergent-like molecules [deoxycholate, sodium dodecyl sulfate], which interact with the OM to increase vesicle formation and remove LPS. Within this process, LPS-containing micelles appear but the obtained OMVs are nearly LPS depleted [70]. On the one hand, the method decreases undesired LPS-inducing innate immune responses; on the other hand, the loss of LPS is related to the loss of bacterial antigens and lipoproteins, which decreases the overall adjuvant effect of OMVs. This balance between beneficial and detrimental effects of LPS removal shows that it is necessary to complement the dOMVs with adjuvants to elicit a useful immune response. Also, dOMVs were shown to form aggregations [111]; this calls for additional research in the field of purification.

### 4.5. Induction by Antimicrobial Peptides [pOMVs]

By supplementing naturally occurring antimicrobial peptides [AMPs] to bacterial cultures, the induction of membrane stress was shown to increase OMV release. Since these host defense peptides are expressed by cells of the innate immune system [all types of granulocytes] as well as from epithelial cells, a response against bacterial antigens does occur. Reactions were shown against cytokines and interleukins such as IL-1ß [116,117,118,119,120] and LPS. Also, there is a well-documented affinity for bacterial membranes. It was shown by Balhuizen et al. [120] that treatment with the AMPs PMAP-36 and CATH-2 of *E. coli* induced the release of OMVs, especially when combined with heat stress. Within the frame of their experiments, strains of *Bordetella bronchiseptica* and *P. aeruginosa* were tested with PMAP-36, CATH-2 and LL-37. While PMAP-36 and CATH-2 induced release of OMVs, the treatment with LL-37 had no effect on vesiculation. The reason may be that LL-37 interacts with LPS in a weak monophasic manner [119,120] compared to PMAP-36 and CATH-2, which interact with LPS in a biphasic manner. It is assumed that the fast vesiculation is a possibility to dispose the membrane regions that are affected by AMPs.

A possible disadvantage may result from the integration of AMPs into the OM of the pOMVs, as shown by Balhuizen et al. [119] for *B. bronchiseptica*. This presence of AMP PMAP-36 led to thermal instability, and it is speculated that this is due to the interaction of positively charged PMAP-36 and phosphatidylglycerol in the pOMV membrane.

### 4.6. Induction by Antibiotics [aOMVs]

Hypervesiculation can also be achieved by the addition of antibiotics [119,120]. There is a large array of functional diversity in antibiotics to induce bacterial stress and hypervesiculation by blocking replication, transcription or translation.

Antibiotics may act on ribosomal subunits to inhibit protein synthesis by blocking the acceptor site for the aminoacyl-tRNA, inhibiting cell wall synthesis [all ß-Lactam antibiotics] or by complexing with terminal L-Lysin and D-Alanin. Also, the inhibition of DNA synthesis by blocking tetrahydrofolate [THF] synthesis or inhibiting topoisomerase II and IV may be applied [121]. Polymyxin B, a peptide-based antibioticum applied to *E. coli* [122] led to increased vesicle production [aOMVs]. Explanations for the inducing process proposed that the peptide-based antibiotics act on the cytoplasmatic membrane structure by disrupting it, leading to aOMV formation. As LPS molecules contain six negatively charged groups [phosphates and carboxylates], this results in a net negative charge of the LPS layer. The addition of polymyxine B alters the membrane stability of the bacterial OM due to the attraction for the positively charged amino groups of the cyclic peptide part of the antibiotic. The following stress-induced destabilization of the membrane results in vesicle formation.

Studies by Yun et al. [122] and Kesavan et al. [123] indicated that the stimulation of *Acinetobacter baumannii* with imipenem and eravacycline resulted in OMV release whereby the vesicles had increased proteases and OMP content. In contrast, the stimulation of *A. baumannii* with tetracycline had no OMV induction effect, which is surprising as both eravacycline and tetracycline bind on the 30S ribosomal subunit. Still, it is well known that even when two antibiotics inhibit the same step in protein synthesis, the inhibition procedure may be due to different molecular mechanisms [41,121,124,125,126]. It has to be noted that aOMVs induced by eravacycline revealed resistance proteins [ATP-binding cassette, transporter proteins] with the disadvantage that antibiotic stimulation with sub-lethal concentrations can probably provoke antibiotic resistance in target cells. Ampicillin-induced aOMVs from *E. coli* resulted in increased amounts of OMP Pal [peptidoglycan-associated lipoprotein] as compared to sOMVs, thus indicating that aOMVs may contain various cargo that may pose problems for standardized vesicle production [126].

### 4.7. Induction by Heat [hOMVs]

As mentioned earlier, environmental stress such as temperature, pressure or nutrient depletion, which a bacterium experiences, may trigger vesiculation [127]. Since von Bergen et al. [29] demonstrated that heat shock treatment of 55 °C increased OMV vesiculation in *Pseudomonas putida*, several research groups started to investigate the heat induction of hOMVs. Similar experiments were conducted with *P. aeruginosa* [128], *B. pertussis* and *B. bronchiseptica* [54]. Even though this strategy to increase vesiculation is not fully established, the heat induction method is promising. In all cases, the presence of antigens on hOMVs was confirmed and a temperature stability up to 40 °C was recorded. All hOMVs elicited immune responses comparable to sOMVs; however, the disadvantage may be the increased hOMV content of LPS, which may have a negative effect on the host’s immune system [128]. A molecular modulation strategy for the immune response is suggested before application can be recommended.

## 5. Bioengineered OMVs

### 5.1. Genetic Manipulation of Bacteria to Increase Vesicle Harvest [gOMVs]

A successful set of methods to obtain both hypervesiculation and specific genetically engineered bacterial strains to produce OMVs with the desired antigens [gOMVs] has been termed GMMA [generalized modules for membrane antigens]. By potentiating or deleting the expression of specific genes, attempts are made to obtain antigen expression on gOMVs to be used as affordable vaccines or vaccine complements. The primordial aim of the GMMA approach was to overcome both the limited OMV yield and to reduce the levels of endotoxicity. Such modules were obtained for *Shigella*, *Salmonella* and *Neisseria* [64,66,127,128,129,130,131,132]. Furthermore, the aim is to obtain vaccines that are fit for purposes in the regions where vaccination may be needed [62,119,120,133,134,135,136]. For this reason, it is useful to combine the expressions of specific antibodies with a high yield of vesicles.

Induced mutations to trigger hypervesiculation are obtained by deleting the *tolR* gene and thereby impacting the proteins of the Tol-Pal system, whereby some of the ToL-Pal proteins may be integrated into the bacterial OM [137,138,139], or by deleting Lpps [140]. Substantial increases in vesiculation were obtained by a mutant of *Shigella flexneri*, Δ *tol R* [141], and a mutant from *S. boydii* [142].

In *N. meningitidis*, the deletion of a lytic transglycolase resulted in hypervesiculation [143] and a *degP* mutant of *E. coli* [144], in which a knock-out of chaperones resulted in misfolded proteins to induce stress-hypervesiculation. Similarly, the deletion of RmpM in *N.meningitidis* indicated the importance of losing anchoring structures to obtain an amplification of OMV production [145,146,147].

Examples for the overexpression of genes inducing increased OMV formation include the expression of deacylase PagL, which increases the curvature of the OM by inverting cone-shaped LPS [73], or overexpression of the OM protease OmpT in *E. coli* [148].

gOMVs have also the potential to deliver antibiotics in order to inhibit the expansion of pathogenic bacteria [149,150]. This special ability is due to the fact that OMVs are able to deliver a variety of biomolecules. Therefore, it was assumed that OMVs also have the potential to be used as natural antibiotic delivery vehicles [149,151].

The aforementioned methods to increase vesiculation and express homologous proteins with antigenic functions have limits when pathogenic bacterial strains are difficult to grow, either because growth rates are very slow or when growth media still need to be modified to obtain optimal yields for VBNC [viable but not culturable] strains; otherwise, the strains in question are resistant to genetic manipulation. To overcome such hurdles, specific proteins can be expressed in heterologous bacterial strains that have fast growth rates and are easy to manipulate genetically. *E. coli* is frequently used as a delivery system for heterologous proteins [152], and genetic manipulation of non-pathogenic bacteria to express specific antigens is recommended. The positive effect of such approaches is a reduced toxicity and the fact that PAMPs, which are present on all bacteria, will support the immune response of the specific heterologous antigen [67,111,118,120,153].

### 5.2. Carrier Protein Fusion with Target Proteins [ClyOMVs]

Promising approaches in presenting heterologous proteins involve a combination of genetic manipulation and conjugation chemistry [154,155]. This methodology is seen as a specific approach within the genetically engineered bacteria to produce heterologous OMVs, which is also true for the glycosylation approach [see below]. Fusion of the gene of a specific target protein with the *clyA* gene for cytolysin A [ClyA] and expressed on OMVs via a plasmid resulted in immune responses on animal models. Huang et al. [149] fused Omp22 of *Acinetobacter baumannii* with ClyA in *E. coli* strain DH5alpha, and the obtained ClyOMVs were used for mice immunization. Challenges of the animals with *A. baumannii* resulted in increased survival compared to controls. Antigens that are fused to ClyA have the advantage of conferring enhanced immunogenicity, because this carrier protein is able to elicit an immune response. It should be mentioned that there are other carrier proteins such as Hbp [hemoglobin protease], fHbp in *N. menigitidis* and ApfA in *Actinobacillus pleuropneumoniae*, indicating the opening of a broad field of investigation [129].

### 5.3. Glycosylation with Heterologous O-Polysaccharides [glyOMVs]

The O antigen of the LPS layer, being the outermost part of this complex and composed of polysaccharides, is highly variable between species with respect to its length, composition and linkages to the core region [74,111,156]. Hence, virulence may vary within species due to variations in O antigen composition. The O antigen is not only a virulence factor but also a B-cell antigen. Genetic engineering opened the possibility to express the genes for an O antigen derived from a pathogenic bacterium in a non-pathogenic strain which then would produce glyOMVs. Many laboratory strains of *E. coli* are devoid of O antigen structures, while lipid A and the core region remain present. It was hypothesized that lipid A with its core region could accept the recombinant O-polysaccharides if the synthesis genes for the O region are supplied [17,157,158], To test this hypothesis, Chen et al. [159] used an O-antigen-deficient *E. coli* strain [JC8031]. Furthermore, this strain was hypervesiculating as the *tolRA* gene was knocked out. By introducing the gene cluster Schu S4 for the synthesis of the O antigen of *Francisella tularensis* in the JC8031 strain, the resulting OMVs expressed the heterologous O glycan on their OM as demonstrated by Western blot analysis. Subsequent vaccination of mice with the glyOMVs resulted in an immune response with high levels of IgG and IgA, which were specific for the heterologous O glycan. Comparable results were obtained for glycoengineered non-pathogenic *E. coli* OMVs by inserting the genes for the biosynthesis of glycans from *Streptococcus pneumoniae* and *Campylibacter jejeuni* [160] Salmonella enterica [161] and *Klebsiella pneumoniae* [54]. Thus far, limitations of the broad application potential [like glycosylation of proteins] of this approach have not yet been detected.

### 5.4. Vesicles with Attenuated Endotoxicity [fmOMVs]

An effective way to obtain attenuated OMVs was achieved by encoding genes that triggered the production of benta-acylated lipid A (see p. 7, 2.1.1.1). Additionally, by transforming *Escherichia coli* W3110 (W3110 Δ*msbB*/Δ*pagP* strain) with the plasmid encoding lipid A 4′phosphatase [pWSK29-LpxF], vesicles were obtained with a modified structure of the polysaccharides of lipid A. The obtained fmOMVs were tested in an influenza vaccine model and significantly reduced TLR 4-stimulating activity was displayed. In comparison to sOMVs, the endotoxicity of fmOMVs was clearly attenuated. Furthermore, a significantly increased T-cell response as well as IgG and IgA levels were recorded when the vaccine antigen was intranasally injected in mice. The obtained vesicles were successfully tested as vaccine replacement against influenza A and B viruses [157], even when challenged with lethal viral doses. Additionally, Lee et al. [157] demonstrated that protection against homologous and heterologous viral challenge was achieved when fmOMVs were co-administered with the influenza vaccine. This is seen as an indication that fmOMVs are promising adjuvants for vaccines.

The advantage of the above-listed types of OMVs and the related production strategy (Table 1) is that different approaches can be combined to improve their applicability as vaccines or vaccine complementation [e.g., dgOMV, agOMVs, lgOMVs, etc.]. This opens a large field of experimentation for the next generation of specific products to cope with the problems of chronic bacterial infections and viral pandemic diseases. Thus, many possibilities remain open for fine tuning of OMVs to be used as vaccines or vaccine complements.

## 6. Mechanisms of OMV Entry into Host Cells

Two major processes are known for epithelial cell penetration; both are driven by endocytic pathways, the clathrin- and the lipid raft-mediated pathways. Among the lipid raft-mediated pathways, there are the membrane regions where the lipid rafts contain caveolin, a membrane protein producing an inlet or bag-like structure in the membrane, supported by dynamin, which has been shown to be involved in the internalization process [57,160]. This entry mode was recorded for OMVs derived from *Haemophilus influenzae* [162], *V. cholerae* [163] and ETEC [164]. Lipid rafts without caveolin were penetrated by OMVs from *P. aeruginosa* [165], *H. influenzae* [160], *C. jejeuni* [162] *Helicobacter pylori* [163,164] and *Moraxella catarrhalis* [166,167]. Clathrin-mediated endocytosis was observed for OMVs from enterohemorrhagic *E. coli* [EHEC] [168,169], *H. pylori* [170,171], *Brucella abortus* and *Aggregibacter actinomycetemcomitans* [166,172]. Observed protrusions from host cell membranes, driven by actin, led to the assumption that such protrusions would not only drive macropinocytosis and phagocytosis but enable bulk uptake of OMVs [109,158], as observed for *P. aeruginosa* [173] and *Legionella pneumophila* [174]. Furthermore, a host factor found on the surface of epithelial cells [bactericidal permeability-increasing protein, BPI] is involved in internalization processes of OMVs, as demonstrated for *N. meningitidis*-derived vesicles [175,176]. An excellent review on pathways involved in OMV entry is provided by O’Donoghue and Krachler [172].

## 7. Discussion

Production and isolation of OMVs for vaccine use is definitely a sensible endeavor because of the vesicles’ property to carry a large array of antigens and to limit pathogen mutations in producing vaccine escape variants. To these ends, the low-cost isolation processes of OMVs compared to vaccine development is an additional attraction for industrial use. A number of experiments and investigations pointed out the importance of retaining the native conformation of antigens on OMVs. *Brucella melitensis*-derived sOMVs were shown to stimulate bone marrow-derived macrophages and interleukin induction [IL-6, IL-10, IL-12 and TNF alpha] in mice [69]. sOMVs from *E. coli* triggered CXCL1 in murine endothelia and a subsequent increased influx of neutrophiles [175]. Immunization with *Vibrio cholerae*-derived sOMVs provoked both immunoglobulin production and protection of mice offspring against *V. cholerae* [19], and Kim et al. [49] showed that this protective effect was due to Th 1 and Th 17 cell responses. Similar observations were made for administered sOMVs from *Shigella flexneri* [177], *Burkholderia pseudomallei* [178] and for *Salmonella enterica* sOMVs in a chicken model where challenges with the pathogen induced interferon gamma expression [179]. Therefore, it is not surprising that many research groups concentrated on the use of OMVs as vaccines, but so far only two types of OMV-based vaccines are currently licensed and in use; these are VA-MENGOC-BC^TM^ and Bexsero, both providing protection against *N. meningitidis* serogroup B strain [MenB]. Both vaccines are good indications for the use of OMVs as vaccine platforms. MENGOC-BC^TM^ is an OMV-based membrane vaccine against N. meningitidis B and C, with the OMVs containing over 100 proteins derived from the meningococcal group B and capsular polysaccharides of group C. Bexsero is an excellent example of a four-component vaccine based on OM proteins and OMVs. It combines recombinant *N. meningitidis* proteins, namely factor H-binding protein [fHbp], Neisseria adhesin protein [NadA], Neisseria heparin-binding protein [NHBA] and OMVs containing the porin protein PorA P1.4 [180,181]. These encouraging examples have stimulated further OMV-based vaccine research concentrating specifically on *N. gonorrhoeae* [182] *Shigella* spp. [183], *Salmonella* spp. [184], *V. cholerae* [19], *Mycobacterium tuberculosis* and *Hemophilus influenza* [185]. Furthermore, OMVs can also be used to deliver antigens against viruses [186,187] and also have utilities beyond fighting pathogens, as shown by the administration of tumor-targeted OMVs [HER2] carrying therapeutic siRNA to mice [188]. A different approach was initiated by Cheng et al. [189], who fused tumor antigens to ClyA carrier protein to obtain T-cell-triggered anti-tumor immunity. In a complementary investigation, the authors fused ClyA with protein catcher systems [SpC or SnC] to capture possible tumor antigens on the OM of *E. coli*-derived OMVs. As this approach led to the detection of multiple tumor antigens, it provides a method to enable cancer patient-specific treatment. Therefore, personalized tumor-specific therapeutic vaccines for cancer will be possible in the near future.

## 8. Outlook

Synthetically produced TLR agonists were recently used as vaccine adjuvants in clinical trials [190]. Monophosphoryl lipid A [MPL], a detoxified form of LPS, is a TLR4 agonist that was approved to be used in vaccines targeting human papilloma virus as well as hepatitis B [190,191]. As MPL was shown to bind TLR4, this resulted in cytokine production, presentation of antigens, AMP migration to T-cell areas and priming of naive T-cells [191]. Further agonists include the non-methylated oligonucleotide CpG, which was identified as a novel adjuvant stimulating TLR 9 and used in cancer vaccine studies; imiquimod as an agonist of TLR 7; and resiquimod, an agonist of TLR 7/TLR 8. All of these molecules were tested in preclinical and clinical studies, resulting in improvements in immunogenicity when added to vaccines [192,193,194,195]. Although significant progress was made in using OMVs as vaccines, the quantities of LPS still pose a problem in that the balance between LPS and immunogenicity without harmful effects to the patient remains pathogen specific. Therefore, new molecules are needed for the modulation of specific immune responses. Indications about the directions of research to be followed up are given by the successful application of human cathelecidin AMP LL-37 [196], chicken cathelecidin CATH-2 [115] and porcine PMAP-36 [119]. It is expected that new synthetic AMPs will be specifically designed for epidemic pathogens in question and will help to combat epidemia and/or pandemia. Finally, it should be considered that the designs of synthetic OMVs consisting of glycophospholipids combined with antigens of interest need to be constructed for the specific pathogens.

In a similar train of thought, it is worth emphasizing that Weyant and co-authors (2023) [197] recently developed an avidin-based vaccine platform by decorating OMVs, whose surfaces were remodeled with biotin-binding proteins and biotinylated antigens. OMVs with such synthetic antigen receptors [SNAP-OMVs] can be decorated with any antigen that is amenable to biotinylation. Mice injected with SNAP-OMVs revealed strong antigen-specific antibody responses.

Essential points to be thought of are [1] the fact that there are differences in the risk of systemic reactogenicity in different age groups [198], as there are differences in the maturation of TLRs between children and adults; and [2] the functions of TLRs [66,127,199,200], indicating species-specific TLR properties.

## 9. Conclusions

Genetically engineering bacteria is definitely a powerful tool to optimize the production of OMVs and their properties to be used as vaccines or vaccine complements. When considering the assembled information on TLRs, PAMPs and related immunogenicity elicited by the administration of OMVs to various hosts, the following needs for OMV-delivering strain optimization can be formulated: [1] Reduction in LPS toxicity is necessary either by modifying these molecules’ biosynthetic pathways or else applying an OMV extraction method that reduces LPS moieties [e.g., detergent extraction, see above dOMVs]; [2] expressing simultaneous antigens combined with an overexpression of the requested antigen for the pathogen in question; [3] retention of antigens that are normally secreted and their exposition on the OM of the OMVs; [4] increasing OMV production to reach hypervesiculation; [5] expressing heterologous antigens either on the OM of the OMVs or in the lumen; [6] inhibiting or removingl components that induce unwanted immune responses; [7] increasing clinical studies to determine which information obtained via studies on animal models may not necessarily be compatible with the human immune system.

All of the developed methods in the field of OMV research thus far are encouraging, and the required optimization can be expected in strain manipulation for the production of fit-for-purpose OMVs to be used as vaccines.

## Figures and Tables

**Table 1:** Types of OMVs with corresponding induction methods, qualitative information on the yields of vesiculation and comments on advantages and disadvantages of each specific OMV induction method.

OMV Types Abbreviation	Induction Strategy	Yield	Advantages	Disadvantages
sOMV	Spontaneous	Very Low	Strong adjuvans	Yield too low for experiments and vaccine use
lOMV	Sonication	High	Fragmentation process easy	Undesirable non-membrane components in OMV
nOMV	Extraction molecules (EDTA)	High	Application easy	Unstable OMV due to removal of Ca++ ions
dOMV	Detergents	High	Increased vesicle formation	Depletion of LPS antigen, adjuvans complementation
pOMV	Antimicrobial peptides (AMP)	High	Hypervesiculation	Thermal instability of OMV
aOMV	Antibiotics	Variable	Diverse use of antibiotics possible	Antibiotic resistance in target cells may occur
hOMV	Heat stress	High	OMVs heat stable up to 40 °C	LPs content increased, harms host immune system
gOMV	Genetic manipulation	Variable	Expression of diverse heterologous OMVs	Related to specific approaches to increase OMV yield +attenuation
clyOMV	Fusion of target protein with Cytolysin A	Variable	Large array of possible applications	Intricate procedure of gene fusion in plasmid and transduction into bacteria
glyOMV	O-polysacch. transfer on core region of non-pathogens	Variable	Large array of possible applications	Variability of O antigen across bacterial species may limit broad applicability
fmOMV	Transformation of strains via lipid A 4’phosphatase gene	Variable	Attenuated endotoxicity. Successfully tested on mice as vaccine replacement	Only preclinically tested with S. enterica combined with dephosphorylase + penta-acylated lipid A

## Data Availability

Not applicable.
